# Risk Factors and Consequences of Cortical Thickness in an Asian Population

**DOI:** 10.1097/MD.0000000000000852

**Published:** 2015-06-12

**Authors:** Saima Hilal, Xu Xin, Seow Li Ang, Chuen Seng Tan, Narayanaswamy Venketasubramanian, Wiro J. Niessen, Henri Vrooman, Tien Yin Wong, Christopher Chen, Mohammad Kamran Ikram

**Affiliations:** From the Memory Ageing and Cognition Centre (MACC), National University Health System (SH, XX, SLA, CC, MKI); Department of Pharmacology, National University of Singapore (SH, XX, SLA, CC, MKI); Saw Swee Hock School of Public Health, National University of Singapore (CST); Raffles Neuroscience Centre, Raffles Hospital, Singapore (NV); Departments of Radiology and Medical Informatics, Erasmus University Medical Center, Rotterdam (WJN, HV); Faculty of Applied Sciences, Delft University of Technology, Delft, The Netherlands (WJN); Singapore Eye Research Institute, Singapore National Eye Center, Singapore (TYW, MKI); Academic Medicine Research Institute, Duke-NUS Graduate Medical School, Singapore (TYW, MKI); Department of Neurology, Brain Center Rudolf Magnus, University Medical Center Utrecht, The Netherlands (MKI).

## Abstract

Cortical thickness has been suggested to be one of the most important markers of cortical atrophy.

In this study, we examined potential risk factors of cortical thickness and its association with cognition in an elderly Asian population from Singapore.

This is a cross-sectional study among 572 Chinese and Malay patients from the ongoing Epidemiology of Dementia in Singapore (EDIS) Study, who underwent comprehensive examinations including neuropsychological testing and brain magnetic resonance imaging (MRI). Cortical thickness (in micrometers) was measured using a model-based automated procedure. Cognitive function was expressed as composite and domain-specific *Z*-scores. Cognitive impairment was categorized into cognitive impairment no dementia (CIND)-mild, CIND-moderate, and dementia in accordance with accepted criteria. Linear regression models were used to examine the association between various risk factors and cortical thickness. With respect to cognition as outcome, both linear (for *Z*-scores) and logistic (for CIND/dementia) regression models were constructed. Initial adjustments were made for age, sex, and education, and subsequently for other cardiovascular risk factors and MRI markers.

Out of 572 included patients, 171 (29.9%) were diagnosed with CIND-mild, 197 (34.4%) with CIND-moderate, and 28 (4.9%) with dementia. Risk factors related to a smaller cortical thickness were increased age, male sex, Malay ethnicity, higher blood glucose, and body mass index levels and presence of lacunar infarcts on MRI. Smaller cortical thickness was associated with CIND moderate/dementia [odds ratio (OR) per standard deviation (SD) decrease: 1.70; 95% confidence interval (CI): 1.19–2.44, *P* = 0.004] and with composite *Z*-score reflecting global cognitive functioning [mean difference per SD decrease: −0.094; 95% CI: −0.159; −0.030, *P* = 0.004]. In particular, smaller cortical thicknesses in the occipital and temporal lobes were related to cognitive impairment. Finally, in terms of specific cognitive domains, the most significant associations were found for executive function, visuoconstruction, and visual memory.

Smaller cortical thickness is significantly associated with cognitive impairment, suggesting a contribution of diffuse cortical atrophy beyond the medial temporal lobe to cognitive function. These findings suggest that cortical thinning is a biomarker of neurodegenerative changes in the brain not only in dementia, but also in its preclinical stages.

## Abstract 2

Supplemental Digital Content is available in the text

Dr Ikram had full access to all of the data in the study and takes responsibility for the integrity of the data and the accuracy of the data analysis. All authors have approved the final article. The sponsors had no role in the design and conduct of the study; collection, management, analysis, and interpretation of the data; preparation, review, or approval of the manuscript; and decision to submit the manuscript for publication.

The Epidemiology of Dementia in Singapore study is supported by the National Medical Research Council (NMRC), Singapore (NMRC/CG/NUHS/2010 [Grant no R-184-006-184-511]). Dr Ikram received additional funding from the Singapore Ministry of Health's National Medical Research Council (NMRC/CSA/038/2013).

## INTRODUCTION

Neurodegeneration – a hallmark of dementia – is characterized by loss of neuronal tissue in both gray and white matter. This brain atrophy is not only seen in clinically manifest Alzheimer disease (AD), but may already be present in the preclinical stages [for which the terms cognitive impairment no dementia (CIND) or mild cognitive impairment (MCI) have been coined].^1–4^ Furthermore, it has been suggested that these brain changes may even be present during normal aging.^5–8^

Recent advances in neuroimaging enable us to assess early age-related brain changes. Of particular interest is cortical thickness, which reflects the width of the cortical gray matter,^9^ and has been proposed to be a reliable marker of brain atrophy.^10^ Previous studies have shown that patients with AD have cortical thinning in frontal, temporal, and parietal regions compared with controls, consistent with pathological patterns of atrophy described in AD.^1,11,12^ In addition, a few studies have suggested that even during the preclinical stages of dementia cortical thinning is associated with worse performance on cognitive tests.^13,14^ Overall, these studies were mainly limited to Caucasian populations, had small sample sizes,^15–17^ and lacked detailed neuropsychological tests.^13,14^

With respect to Asian populations, it has been proposed that – besides neurodegeneration – cerebrovascular disease may play a prominent role in the development of dementia due to the higher prevalence of vascular risk factors among Asians compared with Caucasians.^18–21^ Nevertheless, it remains important to determine the exact role of neurodegeneration in Asian populations, particularly in the preclinical stages of dementia. Thus far, several studies from Korea have shown regional differences in temporo-parietal and prefrontal regions in both AD patients and patients with MCI compared with controls.^22–24^ The association between cortical thickness and cognitive impairment in elderly Asian populations has, however, not been explored extensively. We, therefore, examined whether demographic and cardiovascular risk factors were related to cortical thickness. Furthermore, we examined in an elderly Asian population from Singapore the association of global and lobe-specific cortical thicknesses with cognitive impairment, including preclinical stages of dementia.

## METHODS

### Study Population

The ongoing Epidemiology of Dementia in Singapore (EDIS) study draws participants from the Singapore Epidemiology of Eye Disease (SEED) study, a multiethnic population-based study among persons ages 40–85 years among Chinese (Singapore Chinese Eye Study [SCES]), Malay (Singapore Malay Eye Study [SiMES-2]), and Indians (Singapore Indian Eye Study [SINDI-2]). For this study, we focused on Chinese^25^ and Malay components^26^ of the EDIS Study, as the recruitment of the Indians is still ongoing. In the first phase of the EDIS study, participants ages ≥ 60 years (n = 2666) were screened using the abbreviated mental test and a self-report of progressive forgetfulness. Screen-positive patients (n = 1097) were invited to take part in the second phase of this study, which included an extensive neuropsychological test battery and brain MRI. Of these 1097 participants, 623 agreed to participate in phase II and hence were included in the present study. The details of the study methodology have been described elsewhere.^25^ Ethics approval for EDIS study was obtained from the Singapore Eye Research Institute, and National Healthcare Group Domain-Specific Review Board. The study is conducted in accordance with the Declaration of Helsinki. Written informed consent was obtained in the preferred language of the participants by bilingual study coordinators before their recruitment into the study.

### Demographic and Cardiovascular Factor Assessment

During a personal interview a detailed questionnaire was administered to collect relevant demographic and medical information. Data collection included among others age, sex, race, and education. Previous medical history of hypertension, hyperlipidemia, diabetes mellitus, was also noted and subsequently verified by medical records. Functional status was assessed using The Instrumental Activities of Daily Living (IADL) questionnaire. Clinical assessments included height, weight, and blood pressure. As part of the examinations performed in the SEED cohort, blood was drawn in the nonfasting state to determine full blood count, total cholesterol, glucose, and glycated hemoglobin levels.^25^

Systolic and diastolic blood pressures were measured using a digital automatic blood pressure monitor (OMRON-HEM 7203, Japan) after the patient rested for 5 minutes. Blood pressure was measured twice, 5 minutes apart. Mean of the 2 readings was considered the relevant blood pressure. Hypertension was defined as systolic blood pressure ≥140 mm Hg and/or diastolic blood pressure ≥90 mm Hg, or use of antihypertensive medication. Mean arterial blood pressure was calculated as two-thirds of the diastolic blood pressure plus one-third of the systolic blood pressure. Diabetes mellitus was defined as glycated hemoglobin ≥6.5%, or on medication. Hyperlipidemia is defined as total cholesterol levels ≥4.14 mmol/L, or on medication. Education was categorized into ≤6 years or >6 years of primary education. Smoking was categorized into nonsmokers and smokers (past and current smokers). Body mass index (BMI) was calculated as the weight (kg) divided by the square of the height (meters). IADL score was used as a sum score (higher scores indicate poor functioning; range 0–26). Details of all the study assessments have been described previously.^25^

### Neuroimaging

MRI was performed on a 3 Tesla Siemens Magnetom Trio Tim scanner, using a 32-channel head coil, at the Clinical Imaging Research Centre of the National University of Singapore. Patients with claustrophobia, contraindications for MRI, or those who were unable to tolerate the procedure were excluded. Quantitative MRI data were obtained by automatic segmentation at the Department of Medical Informatics, Erasmus University Medical Center, The Netherlands. For each participant, the following MRI markers were computed:

1. Cortical thickness was calculated using a model-based automated procedure (FreeSurfer, v.5.1.0) on T1-weighted images (TR = 7.2 ms, TE = 3.3 ms, matrix = 256 × 256 × 180 mm^3^). Cortical thickness was measured at each vertex by taking the shortest distance between white matter/gray matter boundary and pial surface.^27^ Whole brain (global) and regional (lobar) averages of cortical thickness were expressed in micrometers. Lobar average was calculated from right and left thicknesses using the parcellation guide on gyral and sulcal structures of cerebral cortex.^27^ Lobar averages were calculated for the frontal, parietal, occipital, temporal, insular, and limbic regions.
2. Total brain and white matter lesions (WML) volumes were also quantified by automatic segmentation. Total brain volume was quantified on Proton density, T1- and T2-weighted images, whereas the WML volume was segmented using the fluid-attenuated inversion recovery (FLAIR) sequence.^28^

Besides these quantitative MRI data, 1 radiologist and 2 clinicians graded the presence of lacunar infarcts and cerebral microbleeds independently and blinded to all neuropsychological and clinical data. Lacunar infarcts were graded on FLAIR and T2 sequences using the STRIVE criteria.^29^ Cerebral microbleeds were defined using Brain Observer Microbleed Scale (BOMBS).^30^ Any disagreement was discussed during weekly consensus meetings attended by study clinicians (neurologists), radiologists, and clinical research fellows, and a final decision was made during this meeting.

### Cognitive Assessment

An extensive neuropsychological battery, which has been previously validated in Singaporean elderly, was administered to assess cognitive function.^25^ The following seven (5 nonmemory and 2 memory) domains were examined;

1. Executive Function (Frontal Assessment Battery, Maze Task),
2. Attention (Digit Span, Visual Memory Span, Auditory Detection),
3. Language (Boston Naming Test, Verbal Fluency),
4. Visuomotor speed (Symbol Digit Modality Test, Digit Cancellation),
5. Visuoconstruction [Weschler Memory Scale – Revised (WMS-R) Visual Reproduction Copy task, Clock Drawing, Weschler Adult Intelligence Scale – Revised (WAIS-R) subtest of Block Design],
6. Verbal Memory (Word List Recall, Story Recall),
7. Visual Memory (Picture Recall and WMS-R Visual Reproduction).

For each participant, raw scores from each individual test within a domain were first transformed to standardized *Z*-scores using the mean and standard deviation [SD] of that test in this cohort. A higher *Z*-score reflected a better performance on that test. Subsequently, for each participant a mean *Z*-score for each domain was calculated by averaging the *Z*-scores of all the individual tests within that domain. These mean *Z*-scores of each domain were then standardized using the mean and SD of that domain-specific mean *Z*-score. Finally, composite *Z*-score reflecting global cognitive functioning was calculated by averaging the 7 domain-specific mean *Z*-scores, which were also standardized using the corresponding mean and SD.

The diagnosis of CIND was determined by clinical judgment and was anchored in the following guidelines, as previously published,^31^ namely, self and/or informant report of problems with cognition without any significant loss of independence in daily activities, and impairment in at least 1 domain of the neuropsychological test battery. Participants were considered to have failed a test if they scored 1.5 SD below education-adjusted cut-off values on each individual test. Failure in at least half of the tests in each domain was considered impairment in that domain. CIND was classified into mild (when ≤2 domains were impaired) and moderate (when >2 domains were impaired). The diagnosis of dementia was made according to the DSM-IV criteria.

## STATISTICAL ANALYSIS

To examine differences in baseline characteristics between included and excluded patients, *χ*^2^ test was used for categorical variables and Student *t* test for continuous variables. Trends in baseline characteristics across different diagnostic groups were examined using analysis of variance (ANOVA) and a *P*-value for the trend test was computed.

Associations of potential demographic and cardiovascular risk factors with global and lobar cortical thicknesses were explored using multiple linear regression models. All continuous variables (age, mean arterial blood pressure, nonfasting blood glucose, total cholesterol, BMI, total intracranial volume) were standardized (by dividing each variable by its SD). For each continuous variable, mean differences in cortical thicknesses were expressed per SD increase/decrease in that variable. Model I was adjusted for age, sex, and education. Subsequently, in the fully adjusted model (Model II), all potential risk factors were included in the same model to determine the independent effect of each potential factor with cortical thickness.

Next, we examined the associations of global and lobar cortical thicknesses with clinical outcomes (CIND and dementia) using logistic regression models [odds ratios (OR) with 95% confidence interval (CI)] and with composite *Z* score using linear regression models [mean difference with 95% CI]. The effect sizes of these associations with cognition were expressed per SD decrease in cortical thickness.

*P*-values <0.05 were considered statistically significant. In view of the multiple tests performed in the lobe-specific analyses, we used Bonferroni correction to obtain a revised statistical significance level of 0.05/6–0.008. Furthermore, we used revised levels of statistical significance for the cognitive domain-specific analyses: 0.05/7–0.007 when analyzing the associations with global cortical thickness, and 0.05/7∗6–0.001 when analyzing the associations with lobar cortical thicknesses. Statistical analysis was performed using standard statistical software (Statistical Package for Social Sciences, SPSS V22, SPSS Inc, Chicago, IL).

## RESULTS

Assessments of study participants were performed from August 12, 2010 to December 21, 2013. Out of 623 patients who participated in phase II, 36 had no MRI scans and 3 had ungradable scans. Furthermore, 12 patients who had a cortical infarct were excluded, as these infarcts may influence the cortical thickness measurements. Supplementary Table 1, http://links.lww.com/MD/A261 presents baseline data of both the included and excluded patients. In brief, excluded patients were likely to be older, were more often Chinese, had lower education, a higher frequency of hypertension and a lower frequency of hyperlipidemia. Out of 572 included patients, 171 (29.9%) were diagnosed with CIND-mild, 197 (34.4%) with CIND-moderate, and 28 (4.9%) with dementia. Table 1 provides baseline characteristics of the included participants according to the different diagnostic groups. In brief, increasing age, female patients, Malay ethnicity, higher proportion of hypertension, diabetes, and hyperlipidemia were related to severity of cognitive impairment. Also, an increasing frequency was observed for several MRI markers. Conversely, a decreasing trend was observed for education, BMI, total intracranial volume, and IADL.

**TABLE 1:**
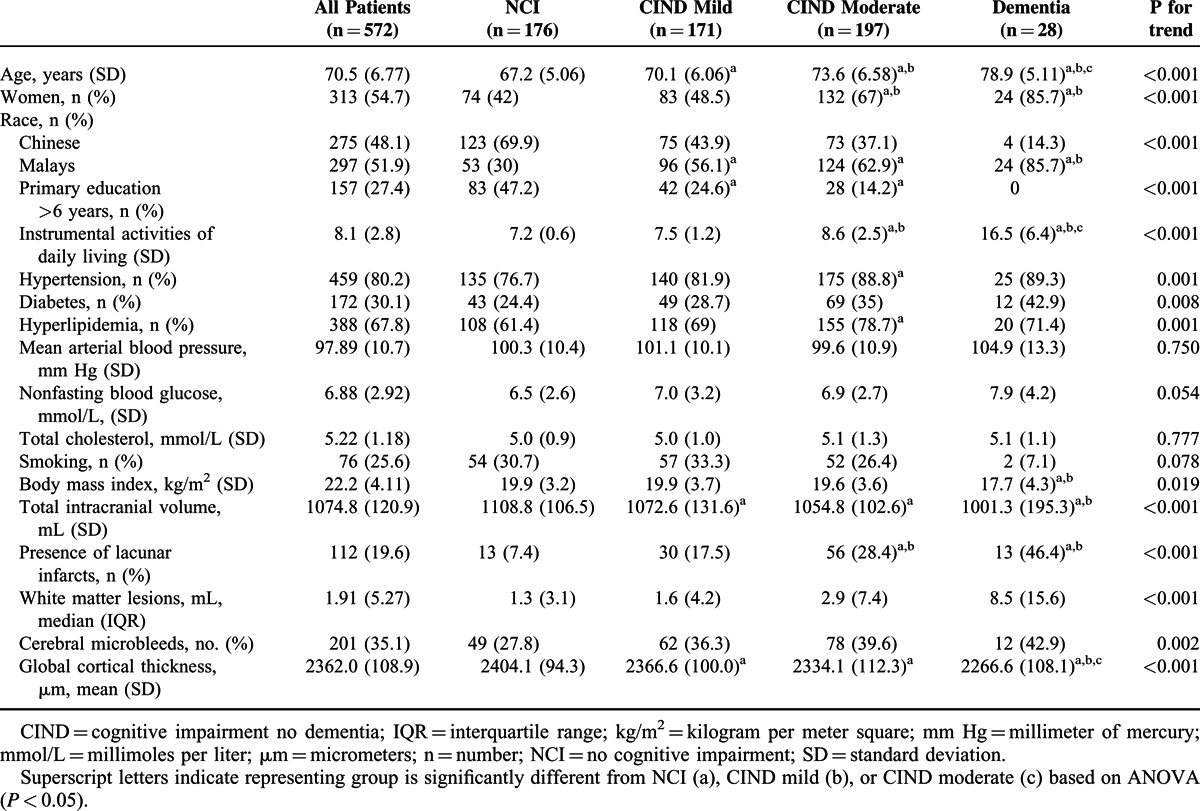
Baseline Characteristics of Study Participants

Table 2 shows the association of potential risk factors with mean global cortical thickness. In fully adjusted models (Model II), the most important risk factors of cortical thickness were: increasing age [mean difference in cortical thickness per SD increase in age: −30.9 μm; 95% CI: −40.2; −21.7; *P* < 0.001], sex [women vs men: 25.4 μm; 95% CI: 2.1; 48.7; *P* = 0.029], Malay ethnicity [Malay vs Chinese: −57.4 μm; 95% CI: −74.5; −40.3; *P* < 0.001], BMI [mean difference per SD increase in BMI: −9.5; 95% CI: −18.1; −0.8; *P* = 0.022], and presence of lacunar infarct [presence vs absence: −25.8; 95% CI: −48.6; −3.1; *P* = 0.034]. A borderline significant association was observed for nonfasting glucose levels [mean difference per SD increase in glucose levels: −8.1 mm; 95% CI: −16.4; 0.3; *P* = 0.059].

**TABLE 2:**
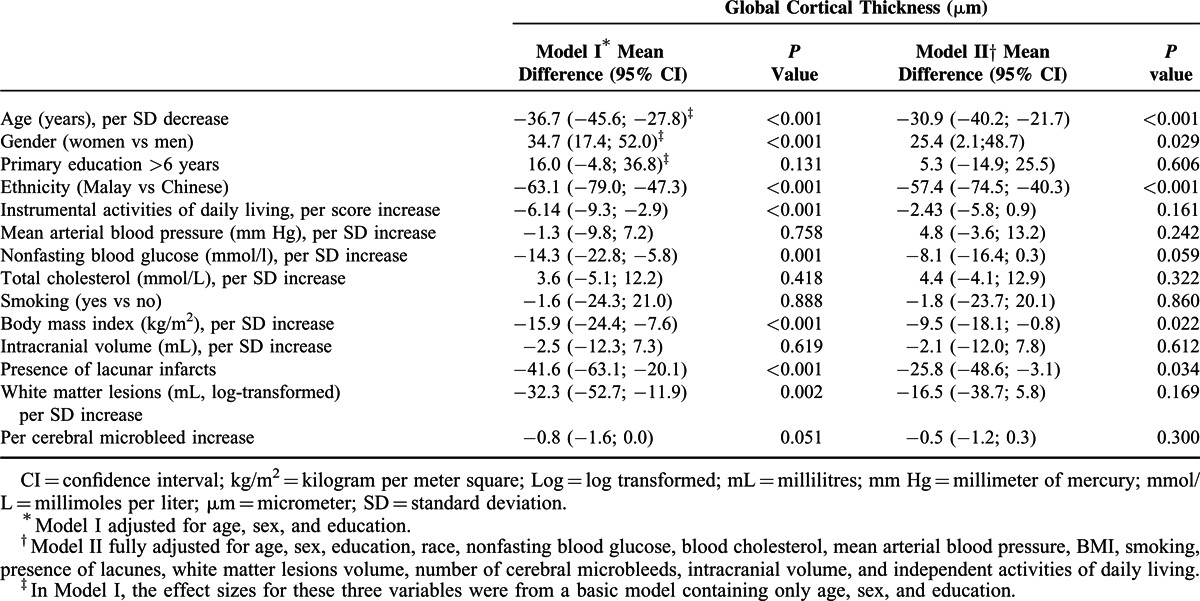
Multivariable Adjusted Associations Between Potential Risk Factors and Global Cortical Thickness (n = 572)

The association between potential risk factors and lobe-specific cortical thickness is presented in supplementary Table 2, http://links.lww.com/MD/A261. After Bonferroni correction, the most consistent associations with smaller cortical thicknesses across the different lobes were found for increasing age and Malay ethnicity. Women had thicker cortical thicknesses in particular in the parietal and temporal lobes. The association between higher BMI and smaller cortical thickness was most prominent in the frontal region [mean difference per SD increase in BMI: −14.7; 95% CI: −23.9; −5.45; *P* = 0.002]. In terms of MRI markers of cerebral small vessel disease, white matter lesions were associated with temporal thinning, whereas increasing number of microbleeds were related to insular thinning.

With respect to clinical outcomes (Table 3), smaller global cortical thickness was significantly associated with CIND moderate/dementia [OR: 1.70; 95% CI: 1.19–2.44; *P* = 0.004]. This association persisted even after excluding 28 dementia cases [OR: 1.69; 95% CI: 1.18–2.43; *P* = 0.004]. Smaller cortical thickness was also related to poorer global cognitive functioning as reflected by the composite *Z*-scores [mean difference composite *Z*-score per SD decrease in cortical thickness: −0.094; 95% CI: −0.159; −0.030, *P* = 0.004]. Lobe-specific analyses showed that these associations were mainly driven by the parietal, occipital, temporal, and limbic lobes. Specifically, the associations with the temporal and occipital lobes remained statistically significant after Bonferroni correction.

**TABLE 3:**
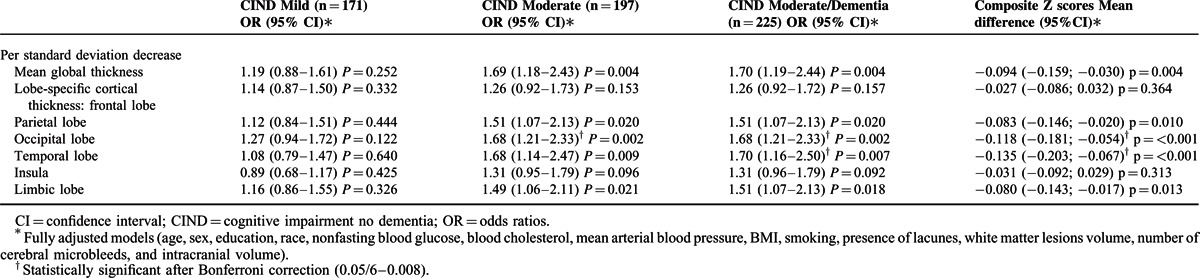
Multivariable-Adjusted Odds Ratios for Clinical Outcomes and Mean Differences in Global Cognitive Functioning per Standard Deviation Decrease in Global and Lobe-Specific Cortical Thicknesses

Finally, in the domain-specific analyses (Table 4), global cortical thickness was related to executive function [mean difference per SD decrease in cortical thickness: −0.129; 95% CI: −0.207; −0.051; *P* = 0.001], visuoconstruction [mean difference per SD decrease in cortical thickness: −0.099; 95% CI: −0.172; −0.027; *P* = 0.007] and visual memory [mean difference per SD decrease in cortical thickness: −0.111; 95% CI: −0.183; −0.039; *P* = 0.003]. In the lobe-specific analyses, the most consistent associations at the nominal significance level of 0.05 were found between the occipital and temporal lobes with the various cognitive domains. However, after applying Bonferroni correction, most of these associations did not remain statistically significant.

**TABLE 4:**
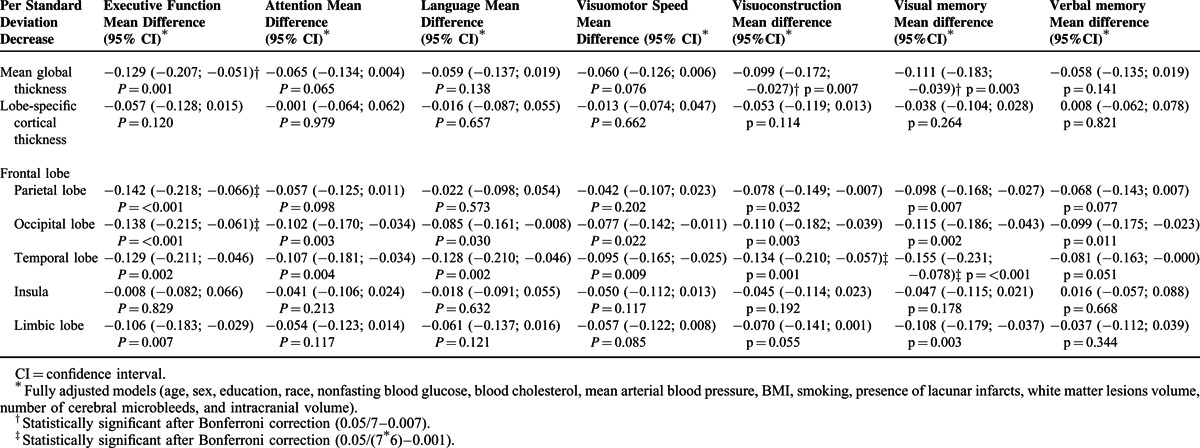
Multivariable-Adjusted Mean Differences in Composite and Domain-Specific Cognitive Function per Standard Deviation Decrease in Global and Lobe-Specific Cortical Thicknesses

## DISCUSSION

In this study, we found that persons with smaller cortical thickness – in particular in the temporal and occipital lobes – were more likely to have cognitive impairment, including the preclinical stages of dementia. More specifically, these persons performed worse on tasks in executive function, visuoconstruction, and visual memory. Finally, the most important risk factors were increasing age, male sex, Malay ethnicity, increased blood glucose, high BMI, and presence of lacunar infarction on MRI.

Several studies reported a smaller global cortical thickness with increasing age.^7,32,33^ Across these studies this effect of age was, however, variable with some reporting the largest decrease in frontal and temporal lobes,^32^ whereas others found the strongest effects in the occipital and parietal regions.^6,34^ The wide age distribution of these studies (ranging from 18 to 82 years) may underlie these differences. Despite these variations, the overall trend – that increasing age was related to smaller cortical thickness – is similar across all these studies, which is further supported by our present findings.

In our study women had relatively thicker cortex compared with men. This sex difference may be related to the protective effect of estrogen on neurodegeneration.^35^ This is in line with other studies reporting similar sex differences in cortical thickness.^27^ In terms of ethnic differences, Malays had a thinner global and lobe-specific cortical thicknesses compared with Chinese. A higher prevalence of vascular risk factors (hypertension, diabetes, and hyperlipidemia) and a higher frequency of Apoε4 carriers have been reported among Malays. These factors may lead to an increased susceptibility to neurodegeneration in Malays and hence may underlie this difference.^36^

With respect to cardiovascular risk factors, we found – in accordance with other studies – that increased blood glucose levels were associated (borderline significantly) with global cortical thinning.^15,27,37^ The mechanisms leading to neurodegeneration are linked to episodes of hypo- and hyperglycemia, alterations to the blood–brain barrier, and increased production of glycated endproducts.^38,39^ Besides glucose levels, an independent association was found for BMI, especially in the frontal lobe. A previous study suggested that adiposity was associated with frontal gray matter atrophy in middle and old aged persons, possibly through increased vascular pathology and reduced blood supply eventually leading to brain atrophy.^40^ Further studies are needed to elucidate the exact mechanisms through which BMI and adiposity are related to atrophy. Finally, several MRI markers of cerebral small vessel disease showed some associations with smaller global and lobe-specific cortical thicknesses, indicating an interaction between cerebrovascular and neurodegenerative processes.^41–43^

With respect to cognition, we found that a smaller global cortical thickness is linked to cognitive impairment, suggesting that diffuse atrophy beyond medial temporal lobe and hippocampus atrophy is already present in the preclinical stages of dementia.^24^ More specifically, thinner cortex in temporal and occipital lobes showed consistent patterns with worse performance in all cognitive domains. Patho-physiologically, the temporal and occipital lobes may show thinning in the early stages of dementia, as these regions are especially susceptible to the toxic effects of neurofibrillary tangles and amyloid plaques,^44,45^ and hence are early sites for these depositions. It has been reported that the burden of these depositions was correlated with the extent of atrophy and reduced metabolism in these regions,^46^ and functionally with cognitive dysfunction. Our present findings suggest that in Asian populations, besides the contribution of cerebrovascular disease, neurodegeneration as reflected by cortical thickness plays an important role in cognitive impairment, including the preclinical stages of dementia.

Limitations of the study include: first, 47.9% of the screened positive patients were excluded from these analyses. Compared with the included participants, these excluded patients were relatively older, less educated, and more likely to have hypertension and hyperlipidemia. Despite this nonparticipation, we however still found significant associations with cortical thickness. Furthermore, these excluded patients might be more cognitively impaired, suggesting that the reported effect sizes in this study might be an underestimation. Second, due to the cross-sectional design of our study the temporal relationship between the presence of cortical thickness and cognitive impairment could not be assessed. Third, due to the small number of cases with dementia, we were not able to examine these cases separately in multivariable models as this resulted in unstable effect sizes and wide confidence intervals. However, the dose–response relationship with the preclinical stages of cognitive impairment suggests that these findings may also be extendable to dementia. Strengths of the study include: patients were selected from a population-based study, extensive neuropsychological tests were used to diagnose cognitive impairment and dementia, and automated and standardized image processing was used to quantify cortical thickness.

In conclusion, persons with smaller cortical thickness – in particular in the temporal and occipital lobes – were more likely to have cognitive impairment, suggesting a contribution of diffuse cortical atrophy beyond the medial-temporal lobe to cognitive function. These findings support the notion that cortical thinning is a biomarker of neurodegenerative changes in the brain not only in dementia, but also in its preclinical stages.
